# Supplementary material for: Analysis of PPARγ Signaling Activity in Psoriasis
Source: Int J Mol Sci. 2021 Aug 10;22(16):8603. doi: 10.3390/ijms22168603 (PMC8395241; doi:10.3390/ijms22168603)
Supplement: Supplementary file 1 [file ijms-22-08603-s001.zip › Supplemental materials_Analysis of PPARg signaling activity in psoriasis/Pathway models/Models images and html files/Interleukin-17 and interleukin-22 signaling in psoriasis/1007186612.html]

Ras GTPase --+> RAF1


# DirectRegulation Ras GTPase --+> RAF1

|  |  |
| --- | --- |
| URN | urn:agi-directregulation:in-out:urn:agi-aopfc:0000465:out:urn:agi-llid:5894:eff=positive:mch=direct%20interaction |
| Connectivity | 2 |
| References | 2784 |
| Effect | positive |
| Mechanism | direct interaction |
| Original # of References | 1444 |

---
